# South West Orthopaedic Club

**Published:** 1983-10

**Authors:** 


### South West Orthopaedic Club

### Meeting at Gloucester on 14th May 1943

### THE PATHOGENESIS OF RHEUMATOID DEFORMITIES OF THE WRIST AND FINGERS
*Pridie Memorial Lecture 1983*
**Raoul Tubiana, Paris**

Professor David Berry, Dean of the Medical School, introduced Professor Raoul Tubiana of Faculte de Medicine, Cochin-Port Royal, Paris. Professor Berry, in his introduction, said that he had met Mr. Pridie many years ago when he had helped provide an implant for a hip joint. He also outlined many of the attributes of Mr. Pridie who above all was an original thinker and an innovator. He then introduced Professor Tubiana, noting his many international honours but also stating that he had a notable collection of modern art and that he was known for his kindness to orthopaedic surgeons in training.

Professor Tubiana then gave the Pridie Memorial Lecture entitled 'The pathogenesis of rheumatoid deformities of the wrist and fingers'. He carefully described and illustrated the anatomy of the wrist, particularly the ligamentous structure which was predominantly on the radial side. He very clearly demonstrated the pathology of rheumatoid arthritis and how this led to subluxation and angulation of the carpus. The disturbed function of the tendons which resulted from the erosion by the rheumatoid tissue both if the ligaments and of the bones of the carpus was clearly illustrated. He then went on to describe the pathology of rheumatoid changes in the metocarpal-phalangeal joints and in the interphalangeal joints. Although the lecture was mainly on the anatomy and pathology of the wrist and hand, Professor Tubiana also outlined some of the treatments which were necessary both in preventing and in curing the deformities.

The lecture was extremely well presented and beautifully illustrated, mainly with line drawings using colour. Prolonged applause followed the lecture and Mr. Merryweather proposed a vote of thanks concluding this with a few sentences in French.

H.R.

### EXPLOSION IN ARUBA
**Hans de Mol Van Otterloo, The Hague**

Dr. Van Otterloo, in a very amusing manner, described an injury to a soldier serving in Aruba when a military rocket accidentally passed through his groin. This had to be sawed in half by an engineer and in doing so he narrowly missed the detonating device. Fortunately neither the sciatic nerve nor the femoral artery were damaged, although the femoral nerve appeared to be divided. This later recovered. The upper end of the femur was shattered. By reconstructing this with bone graft, Dr. Van Otterloo was able to show an excellent result following this unusual and severe injury.

H.R.

### THE GLOUCESTER EXPERIENCE WITH HIP RESURFACING
**W. Lennox, Gloucester**

This paper was a follow up to that presented by Mr. Alan Skirving 2 years ago. At that time 50 patients were available for review 2 years following operation. The period of review has now extended to 4 years and there had been a further 12 patients added who have also been examined 4 years following operation. A total of nine patients had been unsatisfactory and had required further surgery. Five of these were patients with inflammatory arthritis of the hip.

Mr. Lennox felt that this was a worthwhile procedure and even though in patients with inflammatory arthritis problems tended to be greater it was nevertheless an important operation for these patients who particularly needed surgery to the hip. He had modified his technique and in particular the implants over the past 2 years. The acetabular cup has been trimmed thus making it smaller and reducing the chances of inpingement. Reinforced steel mesh has been incorporated in the cement behind the acetabulum and hopefully this will reduce the chances of fracture of the cement. He also now uses as small a femoral head as possible, thus again reducing the amount of friction in the hip joint. He also uses ceramic as opposed to metal femoral caps for the surface of this material is smoother than steel and the particles of polyethylene eroded are likewise smaller. These smaller particles are more easily absorbed by the reticuloendothelial system and are less likely to cause a granuloma.

Mr. Lennox agreed that this operation had a higher incidence of complications than the conventional total replacement arthroplasty, but nevertheless in his hands, the results were sufficiently satisfactory at four years to justify continuing the procedure.

H.R.

### MISTAKES I HAVE MADE IN 40 YEARS OF ORTHOPAEDICS

**H. K. Lucas, Bristol**

Time was limited for Mr. Lucas' paper. In a light-hearted manner he recounted some of his problems, some of which he felt were avoidable, and this talk provided an entertaining conclusion to an enjoyable afternoon.

H.R.

### A PROSPECTIVE STUDY OF THE TREATMENT OF ACROMIO-CLAVICULAR DISLOCATION

**G. C. Bannister, W. A. Wallace, P. G. Stableforth, Dr. M. A. Hutson, Bristol and Nottingham**

*Introduction*
Debate continues as to whether complete acromio-clavicular dislocations should be treated operatively or conservatively.

*Trial*
Over a 27 month period from November 1980, to January 1983, 48 consecutive cases seen at the Bristol Shoulder Clinic and the Nottingham Sports Clinic were randomly allocated to open reduction and internal fixation with a coraco-clavicular AO screw and washer or 2 weeks in a sling. All surgery was performed by, or under the direct supervision of the authors. The screw and washer were removed 6 weeks after fixation.

*Assessment*
Range of movement, level of pain and ability to lift weights were assessed at 6, 12, and 16 weeks with subsequent follow-up at 6 monthly intervals. The present report compares the results at 4 months.

| *At 4 months from injury* | *Conservative* | *Operative* |
|---|---|---|
| Loss of time from work (weeks) | 5 | 11 |
| Loss of time from sport (weeks) | 8 | 16 |
| 100% return of shoulder power (%) | 88 | 85 |
| Residual limitation of movement at 4 months (%) | 12 | 60 |
| Residual pain at 4 months (%) | 30 | 36 |

The results show a significantly earlier return to work and sport and of the range of movement in the conservative group ($<0.05$).

*Results*
The mean time of return to work and sport, and of 100% power are tabulated along with proportion of cases with residual limitation of movement and symptoms 4 months after injury.
Cosmetic assessment revealed that the unreduced dislocation was best tolerated by heavily built patients whilst the best scar resulted from a coronally orientated incision with a subcuticular sutture.

*Failed Management*
In three cases in the operated group the screw failed to engage the coracoid and in one case the screw broke. Three of the conservatively managed cases required surgery for painful subluxation, severe weakness and unacceptable cosmetic disability respectively.

*Re-classification of Acromio-clavicular Dislocation*
Acromio-clavicular dislocations are not a homogeneous group. In five cases in the conservative group the clavicle was displaced superiorly and posteriorly subcutaneously and two of these subsequently required surgery, suggesting that conservative treatment has an unacceptably high failure in severe dislocations.

*Conclusions*
Eighty-five per cent acromio-clavicular dislocations are best treated left unreduced and by early mobilisation. A small group (15%) of severe dislocations with subcutaneous displacement of the clavicle merit operative intervention.

### FEMORAL SHAFT FRACTURES IN CHILDREN, IS THE THOMAS SPLINT REALLY NECESSARY?

**P. Staniforth, Bristol**

Children's femoral shaft fractures always unite, joint stiffness does not occur with immobilisation; overgrowth up to 2 cm occurs during the 2 two years after fracture; remodelling during growth following mal-union due to minor angulations posteriorly and in varus make alignment more important that perfect reduction. The best management is therefore, simple, non-invasive and definitive on the day of injury. Whilst the Thomas knee splint is invaluable as a first aid and a transportation measure, other simple forms of traction produce equally good results.

Gallows traction is indicated in children under 18 kg and some may go home in traction. Simple extension traction with the use of a pillow under the thigh requires the use of sand-bags to control rotation. Equally satisfactory is the addition of the Hamilton Russell knee sling. The more proximal the fracture the less useful is the Thomas splint and the more pillows are required to flex the hip and knee 90° each.

Skeletal traction has complications—pin-track sepsis, knee subluxation and growth plate disorders.

It is indicated only when skin traction fails or cannot be used – e.g. when there is an ipsilateral distal limb injury. Cast braces are not as useful or necessary in children; internal fixation is required only exceptionally (Ziv and Rang, 1983).

Hip spicas have been used since 1898, either as a delayed procedure after preliminary traction, or in recent years put on immediately in the method described by Irani and others in the *American Journal of Bone and Joint Surgery* of 1976. It is suitable for children under 10, with isolated closed fractures.

Assuming home circumstances can be adapted and given adequate equipment for home nursing this technique, if followed precisely, allows the child home within a few days. The results of 27 fractures showed a hospital stay of less than 1 week and satisfactory healing in all. Some rotational problems occurred in common with all previously published series.

In summary, the Thomas splint is therefore but one tool in the successful treatment of these fractures. Simpler and equally effective ways of setting up traction, together with the possibility of out-patient management in plaster casts, should be considered more frequently.

